# Barrier Access Control Using Sensors Platform and Vehicle License Plate Characters Recognition

**DOI:** 10.3390/s19133015

**Published:** 2019-07-09

**Authors:** Farman Ullah, Hafeez Anwar, Iram Shahzadi, Ata Ur Rehman, Shizra Mehmood, Sania Niaz, Khalid Mahmood Awan, Ajmal Khan, Daehan Kwak

**Affiliations:** 1 Department of Electrical & Computer Engineering, COMSATS University Islamabad-Attock Campus, Attock 43600, Pakistan; 2 Department of Computer Sciences, COMSATS University Islamabad-Attock Campus, Attock 43600, Pakistan; 3 Department of Computer Science, Kean University, Union, NJ 07083, USA

**Keywords:** barrier control, sensors platform, vehicle detection, license plate recognition, raspberry-pi, features extraction, machine learning algorithms

## Abstract

The paper proposes a sensors platform to control a barrier that is installed for vehicles entrance. This platform is automatized by image-based license plate recognition of the vehicle. However, in situations where standardized license plates are not used, such image-based recognition becomes non-trivial and challenging due to the variations in license plate background, fonts and deformations. The proposed method first detects the approaching vehicle via ultrasonic sensors and, at the same time, captures its image via a camera installed along with the barrier. From this image, the license plate is automatically extracted and further processed to segment the license plate characters. Finally, these characters are recognized with the help of a standard optical character recognition (OCR) pipeline. The evaluation of the proposed system shows an accuracy of 98% for license plates extraction, 96% for character segmentation and 93% for character recognition.

## 1. Introduction

The security sensitive areas of a country, such as classified defense areas, government buildings and military installations, are under constant surveillance to avoid potential threats. Such surveillance also extends to the vehicles that constantly access these areas. A vast majority of the currently installed systems use barrier gates that are either manually operated [1] or use vehicle identification based on radio frequency identification (RFID) technology [2]. In RFID-based systems, every vehicle has an RFID tag and RFID reader installed at a gate to identify authorized vehicles. Such systems automatize the access control process; however, the installation of RFID tag in each vehicle makes such systems costly. Alternatively, we propose using a combination of a sensors platform and camera system for automatic barrier access control. The approaching vehicle is automatically detected via the ultrasonic sensors while a camera captures the image of the front side of the vehicle. This image is then further processed to extract and recognize the license plate (LP) of vehicle for authorization. Consequently, the barrier is opened only for authorized vehicles.

**Figure 1:**
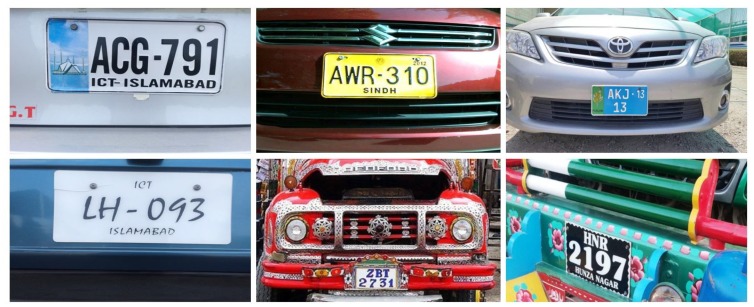
Overview of Pakistani license plates (LPs) with various background, foreground, characters fonts and font-sizes.

An automatic license plate recognition (ALPR) system is instrumental in identifying a vehicle from the image of its LP. As a common rule in various parts of the world, the government issues LPs with fixed aspect ratios, fonts and backgrounds. However, arbitrarily designed LPs is an ever growing problem in countries like Pakistan, where the ALPR becomes a challenging task for a few reasons. First, the position of an LP is not fixed on the front side of the vehicle. Second, there exists a huge variation in the aspect ratios of the LPs. Third, the backgrounds of the LPs vary from on to the other. Finally, variations are also found due to non-uniform font styles and font sizes. Some of these variations are depicted in Figure 1.

A typical ALPR system mainly consists of the following steps [3,4]:

1. Image capturing device acquires an image or extract an image from a video
2. Localization and extraction of license plate in the acquired image
3. Character segmentation and recognition OCR in the extracted LP

The ALPR process begins with LP localization and extraction from the vehicle image. LP localization techniques extract the rectangular bounding box or the text regions directly from the image [5]. Without any prior knowledge about the LP size and its location on vehicle, the entire image must be examined to extract the required LP region. We use a Canny edge detector-based method [6] followed by morphological operations and connected components detection to find the rectangular bounding box around the LP in vehicle image. The next step is the segmentation of the desired LP to extract individual characters for recognition. We propose a segmentation approach for characters that have variations in font size, style, and color. Finally, optical character recognition OCR is used to recognize the letters and digits of the extracted LP. To this end, we adapt the feature-based approach that extracts the features of each individual character. These feature include character contour, zoning of solid binary image character, and a skeleton of thin characters [7,8]. These features are used to train the model of a machine learning algorithm or classifier for character recognition. We evaluate a number of classifiers such as the support vector machine (SVM) [9,10], K-nearest neighbors (KNN) [11,12], artificial neural network (ANN) [13] and Decision Trees [14]. The main contributions of this paper are as follows:

1. Development of a prototype for barrier access control and then deploying it in a real world scenario.
2. An image dataset of challenging number plates commonly used in Pakistan with variations in background, position on vehicle, fonts and font styles.
3. Development of an algorithm for character extraction and segmentation of LPs having different background, position on vehicle, fonts and font styles.
4. An extensive performance evaluation of classifiers for optical character recognition.
5. A performance evaluation of the proposed system on two different hardware environments to select the one which is favorable for real-time application.

The rest of this paper is structured as follows: Section 2 outlines related work; Section 3 explains the proposed methodology; the dataset description, results and performance evaluation are discussed in Section 4; finally, Section 5 concludes the paper and outlines the future directions of the current research.

## 2. Related Work

In this section, we briefly introduce the related work about LP Localization in vehicle images, characters segmentation and characters recognition.

### 2.1. LP Detection and Localization

In an ALPR system, the starting step is LP detection and extraction. If the LP is not properly extracted, then the LP segmentation will be severely affected [15]. As a common practice, an LP has a rectangular shape. However, in the captured vehicle image there may be other rectangular objects such as the headlights. Therefore, for an effective segmentation, the properties and features of an LP such as its area and aspect-ratio, should be known beforehand. Tarabek et al. [16] proposed a connectivity based rectangular bounding-box extraction with fixed properties. The combination of edge detection and morphological operations is used for LP detection and localization [17,18,19,20]. Wang et al. [19] converted the RGB image to HSV color space and proposed a two-stage process for LP localization using color and edge information. Dun et al. [21] proposed an ALPR system for specifically yellow and blue Chinese LPs. A special threshold function was proposed to convert the RGB image to gray to highlight the yellow and blue colors. The transition between the LP background and characters are then used to remove the fake plates and reserve the real plate. In the final step, the accurate location is determined using character size and stroke width. Safaei et al. [22] proposed LP localization based on hierarchical saliency. The proposed algorithm has two steps: in the first step, the algorithm finds the saliency map and then using the connected component analysis detects the LP region. After finding connected components, a Sobel filter and a closing morphological operation is applied. It eliminates many non-number plate regions and then finds the most populated region using $L_{1}$-norm. Its result is then binarized using Otsu’s method. The largest connected component covering the plate number is then cropped from the vehicle image.

### 2.2. Characters Segmentation and Extraction form LP

Character segmentation divides the LP into individual characters and digits. Character segmentation becomes challenging due to multi-color background and foreground of an LP. Tabrizi et al. [23] proposed LP segmentation using morphological operations such as dilation, hole filling, erosion, and characters width and height. Gazcón et al. [24] proposed a bounding box technique and its properties to extract characters from the cropped LP. A Convolutional Neural Network (CNN) based two-stage process is proposed [25] to segment and recognize characters (0–9, A–Z). Tarigan et al. [26] proposed an LP segmentation technique consisting of horizontal character segmentation, connected component labeling, verification and scaling. Horizontal and vertical projections of characters are used to segment the cropped LP [27]. Zheng et al. [28] proposed an improved blob detection algorithm to segment LP characters. The segmentation process consists of three steps: first, character height is estimated using the lower and upper boundaries; character width is estimated; and finally, the character is labeled using the block extraction algorithm.

### 2.3. LP Extracted Characters Recognition

One of the main components of ALPR is the automatic recognition of characters. Chen et al. [29] proposed SIFT based features extraction and matching these features in order to recognize the Chinese characters. A template matching based LP characters’ recognition [30] has been proposed for Arabic characters, to recognize 27 alphanumeric characters (17 alphabets and 10 numeric) of fixed size 50×25. A tesseract OCR engine [31] with modification is used in Reference [28] for LP characters’ recognition. Tabrizi et al. [23] proposed a hybrid approach of k-nearest neighbor (KNN) and multi-class support vector machine (SVM) for Iranian LP recognition. First, the KNN classifies the characters using the structural, horizontal and vertical features. Then the SVM classifier is applied to the zoning features. Gazcón et al. [24] compared the proposed intelligent template matching (ITM) with the artificial neural network (ANN). Compared to the traditional template matching technique the ITM constructs trees of the character’s skeleton. These trees are used to compare with the tree obtained from the testing character skeleton. ITM showed higher accuracy and also minimized the recognition time. Wang et al. [32] proposed LP detection and recognition simultaneously in a single forward pass by using a deep neural network algorithm. In the first step of this algorithm, a number of convolutional layers are used to extract and discriminate the features of LP. After this, the proposed network detects the objects on a LP. This technique takes the low level convolutional features and generates a set of bounding boxes. In the last step, a bidirectional recurrent neural network (BRNN) with Connectionist Temporal Classification recognizes the LP characters. Björklund [33] proposed an ALPR system trained on synthetic data that has varying pose conditions and illumination levels and showed precision and recall of 93%. Table 1 presents the overall literature review of LP detection, LP region of interest extraction, characters’ segmentation, and character recognition.

## 3. Proposed System

**Figure 2:**
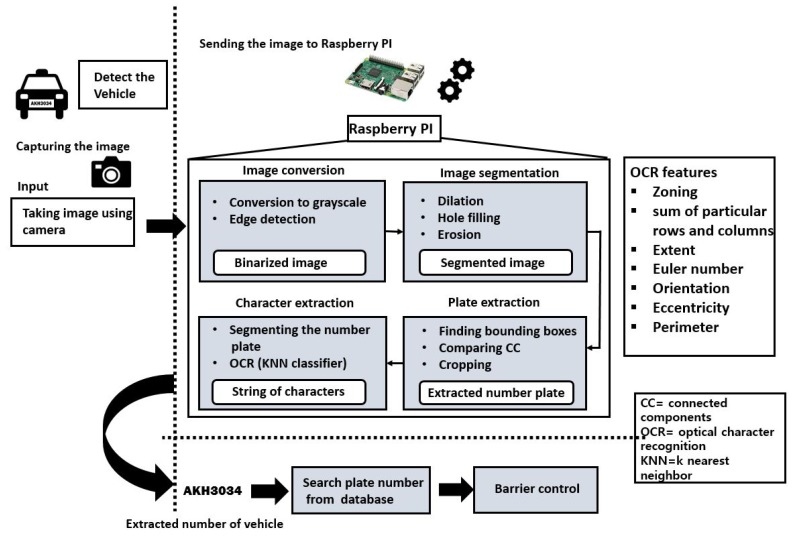
The proposed architecture of barrier access control for vehicle entrance using sensors platform and an image-based LP recognition.

**Figure 3:**
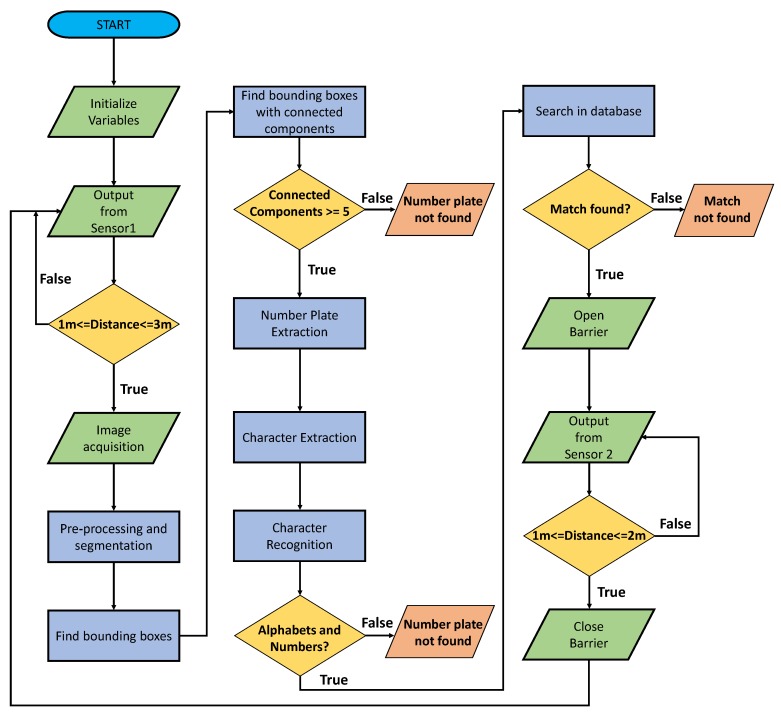
Algorithm flow chart of the proposed smart access control for vehicle entrance using sensors platform and an image-based LP recognition.

This section explains the proposed architecture including the main functions from vehicle detection to the barrier control mechanism. Figure 2 illustrates the block diagram of the proposed system while Figure 3 depicts the algorithm flowchart of the proposed system. Following are the main steps.

1. Vehicle arrival detection and image acquisition
2. Image pre-processing and edge image generation
3. Image segmentation based on detected edges
4. LP extraction via the count of connected components
5. Character segmentation and features calculation
6. Optical character recognition
7. Vehicle authorization and barrier control system

These steps are further explained in the following subsections.

### 3.1. Vehicle Arrival Detection and Image Acquisition

**Figure 4:**
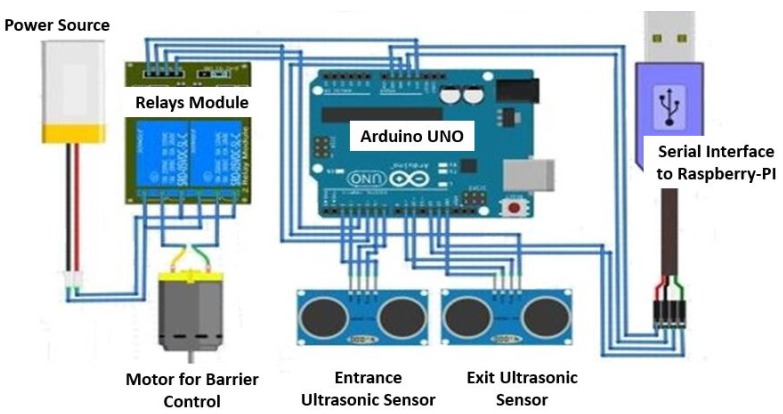
Hardware architecture of an ultrasonic sensors-based vehicle entrance and exit detection.

Figure 4 shows the proposed hardware architecture for barrier access entrance control. Ultrasonic sensors installed at the barrier detect the approaching vehicle. The sensor emits 8-pulses of 40 KHz for 10 μs and listens to the echo signal for 100 μs to 36 ms. Using $S=\frac{Vt}{2}$, we find the distance between barrier and the vehicle where *S* is the distance, *V* is the speed of sound: .034 m/μs and *t* is the time in μs for transmission and its echo signal. The camera is only activated for image acquisition when the ultrasonic sensors detect the vehicle in a specific range of distance which is set from 1 to 3 m. As a common practice on gate entrances, a lane is built for the entering vehicle so that they are almost straight when the image is taken by the camera. Due to this reason, the image of the entering vehicle is taken with negligible rotations.

### 3.2. Image Pre-Processing and Edge Image Generation

In the proposed ALPR method, we convert the captured image into grayscale. It reduces the processing complexity and processing time and is robust to color changes due to different lighting conditions. A canny edge detector is applied to this image to detect all the edges. The Canny edge detector is a combination of a Gaussian filter for smoothing and a Sobel filter for edge detection. Equation (1) shows the Gaussian filter that suppresses the noise in an image with σ as the standard deviation of the Gaussian filter.

(1) $$G\left(x,y\right)=\frac{1}{2\pi \sigma^{2}}e(-\frac{x^{2}+y^{2}}{2\sigma^{2}})$$

(2) $$S_{x}=\left[\begin{array}{ccc} +1 & 0 & -1\\ +2 & 0 & -2\\ +1 & 0 & -1 \end{array}\right]\;S_{y}=\left[\begin{array}{ccc} +1 & +2 & +1\\ 0 & 0 & 0\\ -1 & -2 & -1 \end{array}\right]$$

(3) $$\left|S\right|=\sqrt{{S_{x}}^{2}+{S_{y}}^{2}}$$

(4) $$∠S=\tan^{-1}\left(\frac{|S_{y}|}{|S_{x}|}\right)$$

After the Gaussian filter, we apply the Sobel masks [37] to detect the horizontal and vertical edges as shown by Equation (2). Equations (3) and (4) show the magnitude and direction of the Sobel gradient respectively. Considering the pixel magnitude, direction, non-maximum suppression and thresholds, the pixel is marked as an edge if its magnitude is greater than the threshold in the gradient direction. Finally, at this stage, we get an edge segmented image.

### 3.3. Image Segmentation Based on Detected Edges via Morphological Operations

On the generated edge image, we perform various morphological operations such as dilation, horizontal erosion, vertical erosion and hole filling. Dilation adds the pixels to the boundary of edges to complete the boundary and increases the efficiency of LP extraction. Mathematically, Equation (5) shows the dilation.

(5) $$I_{\mathrm{dilated}}=I⊕B=\left\{z|{\hat{\left(B\right)}}_{z}\cap I\neq \emptyset \right\}$$

where I is edge segmented image and B is structure element. After dilation, we filled the closed boundaries and remove unnecessary parts of the image without affecting the LP area. A hole filling technique is used for this purpose and its mathematical expression is given by Equation (6).

(6) $$I_{\mathrm{holefilled}}=X_{k}=\left(X_{\left(k-1\right)}⊕B\right)\cap I_{\mathrm{dilated}}^{c}$$

We use vertical and horizontal erosion to remove those pixels, which makes it difficult to extract the LP. All the unnecessary lines and parts connected to the LP area create problems for the LP extraction. Equation (7) shows the mathematical expression used for erosion.

(7) $$I_{\mathrm{eroded}}=I_{\mathrm{holefilled}}⊖B=\left\{z|{\left(B\right)}_{z}\in I_{\mathrm{eroded}}\right\}$$

### 3.4. LP Extraction via the Count of Connected Components

We find the 8-connectivity components based rectangular bounding box objects in the eroded image. In addition to the LP, there are other rectangular objects such as headlights, radiators, grille and bumper. Therefore, it is likely that these objects are also segmented along with the LP. Due to this reason, we use the count of connected components in each segment as a clue to differentiate between the LP and other rectangular objects. To this end, for a segment to be considered an LP, the number of objects inside that segment should be more than five. This is due to the fact that the Pakistani LP consists of at least five characters as shown in Figure 3. Once the mask of the LP is generated in this way, it is used to extract the LP from the RGB image.

### 3.5. Characters Segmentation from LP Segment and Features Calculation

**Figure 5:**
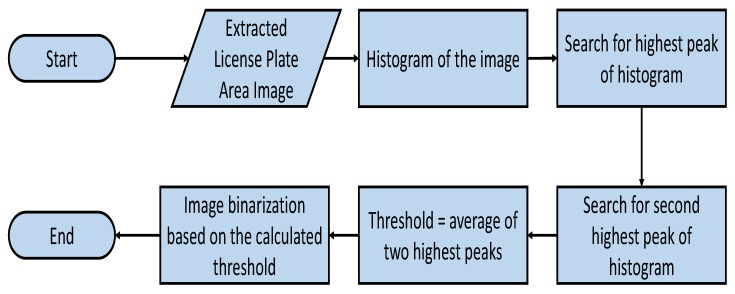
Thresholding algorithm to convert the LP grayscale image to binary.

Once the LP region is extracted, character segmentation is employed to extract the LP characters. For this purpose, as a first step, the LP region is binarized using the algorithm shown in Figure 5. First, we calculate the intensity histogram of the LP region image and then find the two highest peaks in this histogram. We considered the two highest peak because the LP mostly consists of two colors, that is, the LP background color and the characters’ color. The threshold is the average value of these two peaks.An LP grayscale image is then binarized using this threshold. We extract characters from the binary image using 8-connectivity, considering a character height of 30 to 90, a width of 10 to 40 and an area of 700 to 800 pixels.

In this paper, we focus on the features-based approach for character recognition. We extract the following features of a character.

**Figure 6:**
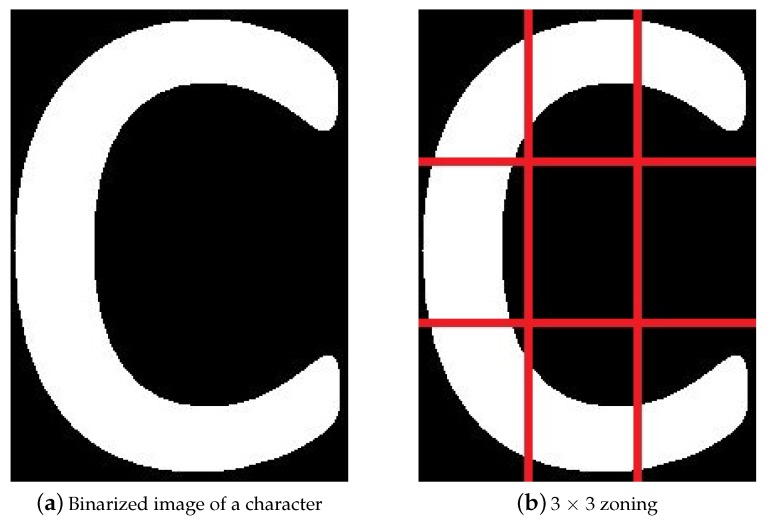
Overview of zoning of an image into sub-images.

- **Zoning**: It divides the character image into various sub-images. Figure 6 shows the overview of zoning a character image into 3×3 sub-images. The white pixels are summed in each sub-image and become a feature.
  Mathematical it can be calculated by Equation (8).
  (8) $$zoning_{feature}=\sum_{i=1}^{M}\sum_{j=1}^{N}\mathrm{subimage}\left(i,j\right)$$
  where M×N is the size of sub-image. We considered a character 42×24 size of image and then divided it into nine sub-images of 14×8 each.
- **Perimeter:** The set of interior boundary pixels of a connected component (character image (**C**)) [33]. We considered 8-connectivity to find the perimeter. Equation (9) finds the perimeter of a character.
  (9) $$Permiter_{8}=\left\{\left(a,b\right)\in C|N_{4}\left(a,b\right)-C\neq \emptyset \right\}$$
  where (a,b) is the pixel location.
- **Extent:** It is the ratio of white pixels in an image to the total number of pixel in the binary image. Equation (10) finds the Extent value of a character image.
  (10) $$Extent=\frac{Number\_of\_WhitePixels}{Total\_Pixels\_Image}$$
- **Euler Number:** Euler number is the topology measure of an image. It is the number of objects in an image minus the number of holes in the image. Equation (11) finds the Euler number:
  (11) Euler_number=1−number_of_holes
- **Particular Rows and Columns Pixels Summation:** In the paper, we consider some particular rows and columns to add their pixels. That particular row or column pixels summation is considered as a feature. Equation (12) finds the sum of a particular row *i*.
  (12) $$sum\_row\_i=\sum_{col=1}^{TotalCol}C\left(i,col\right)$$
  where TotalCol shows the number of columns in the character image **C**. We considered the summation of rows third, fifteen, twenty-seven and thirty-seven as features. The summation of the column is given by Equation (13).
  (13) $$sum\_column\_j=\sum_{r=1}^{R}C\left(r,j\right)$$
  where *R* is rows in character image **C** and we find the summation of second, twelve and seventeen columns.
- **Eccentricity:** Finds how close an object is to being a circle. It is ratio of the linear eccentricity to the semi-major axis.
- **Orientation:** The major axis of an ellipse around the object and then finding the angle which the major axis made with the x-axis.

### 3.6. Optical Character Recognition of LP Characters

**Figure 7:**
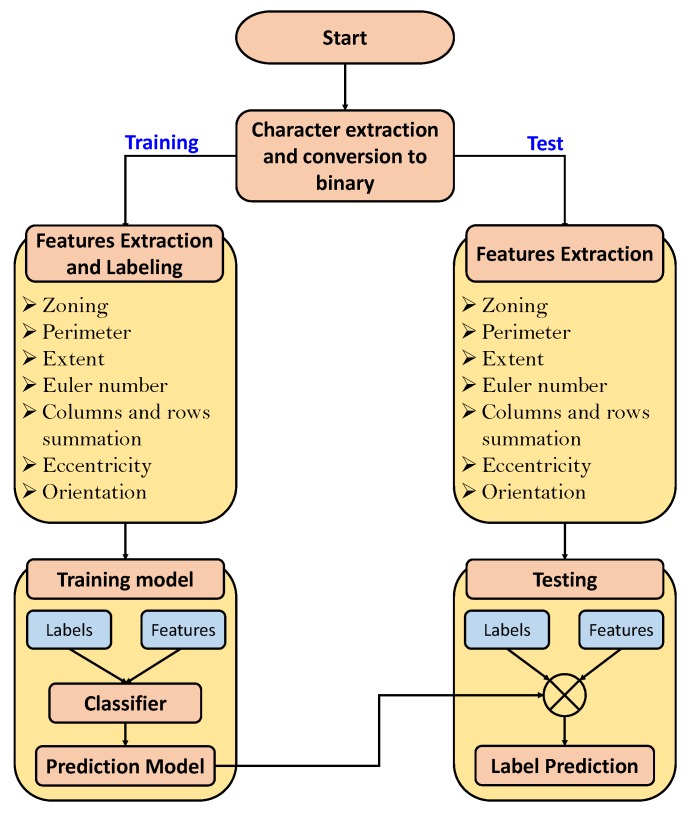
An overview of LP characters recognition process.

In this paper, we evaluated various supervised learning algorithms (classifiers) to recognize characters on the LP. Figure 7 shows the process of LP character recognition. As a first step, these characters are manually extracted from the images. The aforementioned features of each extracted character are calculated in order to represent each of them in a single feature vector of length 21. A given classifier is then trained on these features. For testing, the proposed extraction algorithm first extracts the LP characters automatically while the trained classifier recognizes the characters by predicting their labels. We used KNN, Decision Trees, Random Forest, SVM, and ANN for LP character recognition.

### 3.7. Vehicle Authorization and Barrier Control System

**Figure 8:**
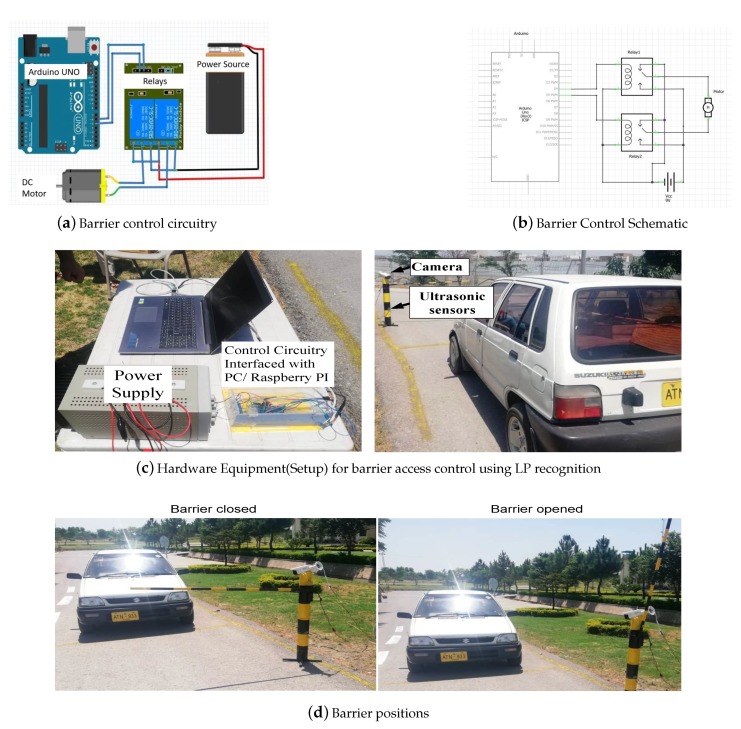
Hardware Setup and mechanism of barrier system control using DC motor.

The real-time system for vehicle detection and authorization is implemented on a Raspberry Pi. The ultrasonic sensors interfaced to the Raspberry Pi detect the approaching vehicle on entrance. The LP of this vehicle is then verified using its image. If the vehicle is permitted then the Raspberry Pi sends a command to open the barrier. Figure 8 shows the circuit, schematic, hardware setup and access mechanism of the barrier control system. Figure 8c shows the real-time hardware setup used to detect the vehicle, recognize the LP and control the barrier position. A camera and two ultrasonic sensors installed on the barrier are also shown. The front ultrasonic sensor detects the vehicle at the entrance and the rear ultrasonic sensor detects the exited vehicle. The barrier control circuitry is interfaced using the RS-232 serial port to the LP processing system. We used a DC motor [38] to control the barrier access that rotates between 0° and 360°. We used 90° and +180° for closed and open barrier systems respectively, as shown in Figure 8d. The motor rotates the barrier bar from open to closing when the first relay is active and second is de-active and vice-versa. In real implementation, we used the DC motor rated 24 V of high torque which can easily move a barrier bar that weighs upto 8 kg. However, in a PC based simulation, we used 9 V to simulate the controlling of the motor.

## 4. Results & Discussion

The proposed system is implemented on the following frameworks.

1. PC(Intel(R), Core(TM) i3-4010U CPU 1.70GHz,RAM: 4.00GB) running Matlab(R2013a, 64-bits) and interfaced the Arduino using an RS232 serial port for the barrier control system. Matlab programming and Arduino C-based code are used to implement the system
2. Raspberry Pi- system on chip single board computer with 1.4 GHz 64-bits quad-core processor and interfaced the Arduino using RS232 serial port for the barrier control system. Python 3, OpenCV 3.4.0, and Arduino C-based code is used to implement the system.

Table 2 shows the details of the acquired dataset used as training and test images. Images were taken with a camera in daylight conditions.

### 4.1. Results of Pre-Processing, Edge Detection, and LP Area of Interest Extraction

**Figure 9:**
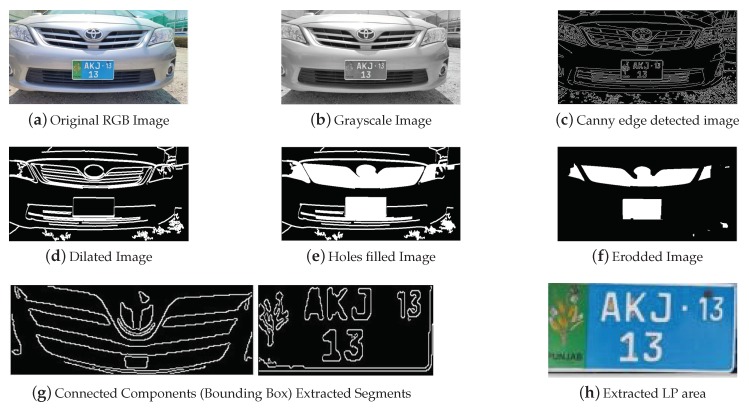
Results of pre-processing, edge detection, morphological operations, connected components extraction and final LP area extraction.

Figure 9 shows the qualitative results of pre-processing before LP extraction. Figure 9a shows the original RGB captured image and the resized image when detected by the ultrasonic sensor in the specified range. Figure 9b shows the RGB image converted to grayscale and Figure 9c shows the detected edges in the image via Canny edge detector. Figure 9d–f shows various morphological operations applied to the edge image. A 5 × 1 structure element of Dilation enlarges the edges. Hole filling fills the connected objects and erosion removes the pixels on object boundaries and the single pixel objects (lines). Connected components based segments are extracted using a constraint of connected objects on a segment as shown in Figure 9g. Finally, Figure 9h shows the extracted LP area of interest.

**Figure 10:**
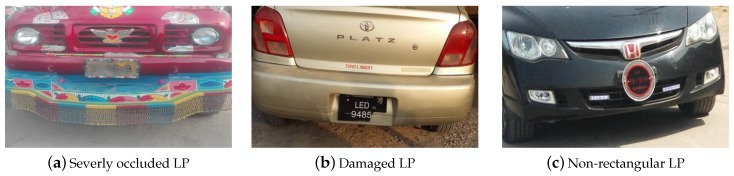
Vehicle images where LPs are not correctly segmented due to various reasons.

For 500 images, the LP extraction accuracy of the proposed method is 98%. Figure 10 shows images where the LP is not correctly extracted due to various reasons such as character occlusion due to dirt, non-rectangular LP and broken LP.

### 4.2. Results of LP Characters Segmentation

**Figure 11:**
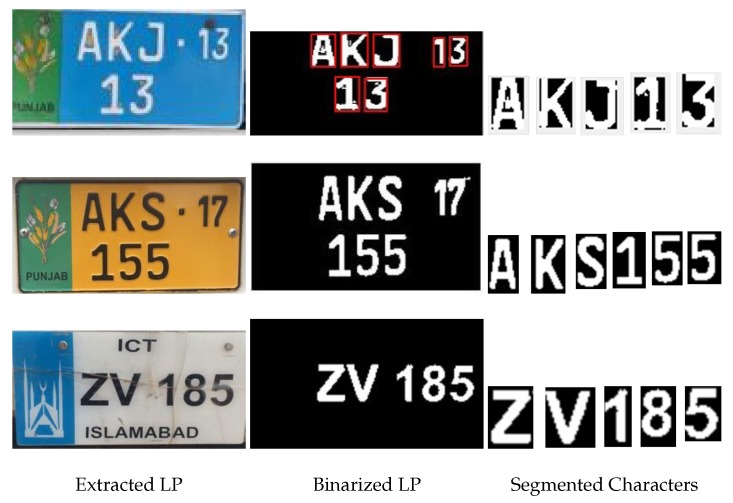
LP extraction, binarization and character segmentation.

The step-by-step result of the LP segmentation and character recognition are visually shown in Figure 11. The variations in the LP background, font sizes and styles of the characters’ positions can be observed in the different types of LPs. There are also additional numbers and characters in the LPs. The proposed method clearly shows its robustness to such challenges and extracts the bounding boxes that enclose only those characters that belong to the license plate’s number. The LP character segmentation of the proposed method for an image dataset of 500 images is 96%.

### 4.3. Results of LP Characters Recognition

**Figure 12:**
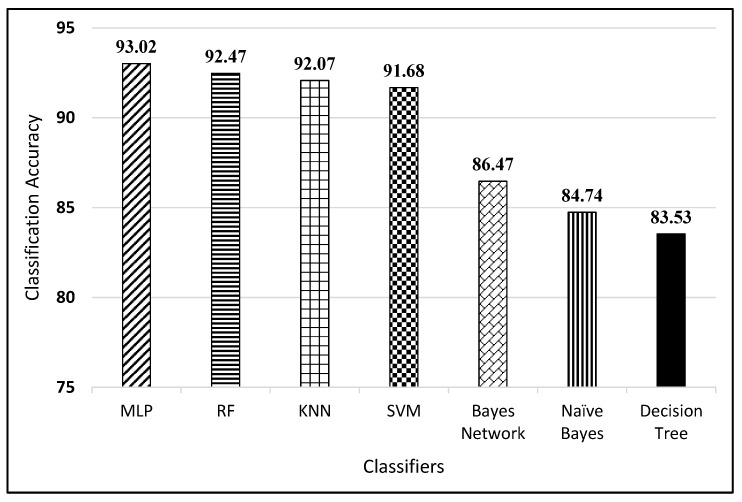
Classifiers accuracy performance comparison for OCR.

We assigned class labels 0–9 to the digits and 10–35 for alphabets A–Z to recognize the LP characters. A total of 3643 characters were extracted from images of computerized and handwritten LPs. The aforementioned features were calculated for each of the extracted characters. In the current setting, we evaluated various classifiers such as KNN, Naive Bayes, Bayes Network, SVM using linear kernel, MLP, Decision Tree and RF for LP character recognition. The classifiers training and testing was done using 10-fold cross validation where a given dataset is split into 90% training set and 10% test set. The performances of classifiers were compared with respect to their classification accuracy, true positive rates, false positive rates, precision, recall, F-measure and ROC area. Figure 12 shows the classification accuracies of all the classifiers on the given dataset. Table 3 shows the detailed comparison of classifiers with respect to other metrics.

Table 4 and Table 5 show the confusion matrix of OCR using the KNN and MLP algorithms, respectively. The characters on the LPs are handwritten and computerized. Mostly, the handwritten characters such as 0 have higher similarity with *O* and also *Q* with 0. 5 and *S*, and *M* and *N* have higher similarity. If they are computerized based *O*, *Q*w and 0 are used it will increase the recognition accuracy.

The accuracy of MLP, KNN, SVM and RF are close to each other. Therefore, we implemented the KNN algorithm for real-time ALPR both on Matlab and Raspberry Pi based proposed systems. Table 6 shows the time analysis of the proposed system, both implemented using a PC with Matlab and Raspberry Pi with Python and OpenCV library. It depicts the time from vehicle detection to LP number recognition and barrier access control opening. The time consuming part of the proposed system is the pre-processing-LP localization and recognition of LP characters. We compared the timing for 100-LPs that had 5-characters, 6-characters, and 7-characters, respectively. The Raspberry Pi based system had the lowest computation time, a small size and low power requirements. It can be easily installed in the constraint area for vehicle detection and control access to a restricted area.

## 5. Conclusions

We presented a robust, accurate, industrial barrier access control system using a sensor platform and vehicle license plate recognition. The proposed system automatically detects a vehicle at an entrance via ultrasonic sensors and then recognizes it by image-based recognition of its license plate, which can have various backgrounds, fonts and font styles. To this end, a performance evaluation of various classifiers was carried out to find out that which had the best recognition rate. Lastly, the proposed system was implemented both on a PC running Matlab and on a Raspberry Pi (system on chip) running Python with OpenCV. The Raspberry Pi-based system had low computational time, a smaller size, and low power consumption, due to which it was used in the real-time application. In future, we are working to increase the dataset of handwritten LP characters to improve accuracy and laser beam-based vehicle detection to increase the detection range.

## Figures and Tables

**Table 1:** Literature summary of LP detection, extraction, characters segmentation, and characters recognition.

Ref.	Methodology	Character Language (0–9, A–Z)	Dataset Size	Platform	Plate Localization	Character Segmentation	Character Recognition	Processing Time
[28]	Plate Detection:Viola and jones algorithm, Character Segmentation: Blob detection, Characters Recognition: OCR	(0–9) (A–Z)	Testing dataset (160) Training dataset (300)	PC with Pentium 2.8 GHz CPU	96.4%	98.2%	99%	0.1 s
[26]	Recognition: Genetic algorithms	(0–9) (A–Z)	220 Images	PC	N/A	N/A	85.97%	0.2 s
[23]	Characters Extraction & Segmentation: Morphological operations, Recognition: KNN and SVM	Persian characters	257 Images	PC	96.01%	95.24%	97.03%	N/A
[34]	Segmentation: Otsu’s method, Character Extraction:Vertical projection, Recognition: Backward propagation Neural Network (BP-ANN)	(0–9) (A–Z)	Training: 2700 & Testing: 354 characters	CPU i3 2.9G and 2G memory	N/A	N/A	93.5%	N/A
[35]	Detection, Extraction, and Recognition: Convolutional Neural Network (CNN)	(0–9) (A–Z) Excluding ‘O’	Testing USA (328) Europe (550)	PC	99.0% USA 93.64% Europe	N/A	93.445% USA 94.54% Europe	N/A
[36]	Plate Detection:Modified visual attention model, Segmentation: Vertical and horizontal projection, Recognition:CNN and SVM	Chinese (0–9) (A–Z)	Testing: Chinese (620), 0–9 and A–Z (680): Training Chinese (930), 0-9 and A-Z (1020)	PC	98.9%	N/A	98.3% Chinese characters 99.1% (numerals and alphabets)	0.14 s
[24]	Through searching rectangles (plate extraction) Bounding Box technique (character extraction) ANN and ITM (character recognition)	(0–9) (A–Z)	Testing data (73)	Pentium Dual core, 1.73 GHz, 2 GB RAM	N/A	N/A	99.09% (ANN) 91.1% (ITM)	0.5 ms (ANN) 0.75 ms (ITM)
[27]	YOLO detector (vehicle and LP detection) CNN (character segmentation and recognition)	(0–9) (A–Z)	Data set (4500)	NVIDIA Titan XP GPU	100%	99.75%	97.83%	28.3 ms

**Table 2:** Dataset description for LP extraction, characters segmentation and recognition.

Sequence No.	Description	No. of Images
1	Images for LP extraction	500
2	Images for characters segmentation	500
3	Characters (0–9) & (A–Z)	3643
4	Features vector dimension	21

**Table 3:** Comparison of performance metrics (TPR, FPR, Precision, Recall, *F*-measure, and ROCArea) of classification algorithms for OCR.

$\frac{\mathrm{Classifiers}}{\mathrm{Parameters}}$	TPR	FPR	Precision	Recall	F-measure	ROC Area
KNN	0.921	0.003	0.921	0.921	0.92	0.959
Bayes Network	0.865	0.005	0.868	0.865	0.865	0.994
Naive Bayes	0.847	0.006	0.859	0.847	0.85	0.99
SVM	0.917	0.004	0.913	0.929	0.916	0.956
**MLP**	**0.93**	**0.002**	**0.931**	**0.93**	**0.93**	**0.995**
Decision Tree	0.835	0.007	0.832	0.835	0.833	0.93
RF	0.93	0.004	0.93	0.925	0.926	0.965

**Table 4:** Confusion matrix of OCR using KNN classifier algorithm.

$\frac{\mathrm{Actual}\;\mathrm{Class}}{\mathrm{Predicted}\;\mathrm{Class}}$	0	1	2	3	4	5	6	7	8	9	A	B	C	D	E	F	G	H	I	J	K	L	M	N	O	P	Q	R	S	T	U	V	W	X	Y	Z
0	148	0	0	0	0	0	0	0	0	0	0	0	0	0	0	0	0	0	0	0	0	0	0	0	6	0	0	0	0	0	0	0	0	0	0	0
1	0	156	0	0	0	0	0	2	0	0	0	0	0	0	0	0	0	0	6	0	0	0	0	0	0	0	0	0	0	0	0	0	0	1	0	0
2	0	1	223	3	0	0	0	0	1	1	0	0	0	0	1	0	0	0	0	1	0	0	0	0	0	0	0	0	2	0	0	0	0	1	0	3
3	0	1	3	195	0	4	0	0	0	0	0	0	0	0	0	0	0	0	0	0	0	0	0	0	0	0	0	0	1	0	0	0	0	0	0	0
4	0	0	0	1	212	0	1	0	0	1	2	0	0	0	0	0	0	0	0	0	0	1	0	0	0	0	0	0	0	0	0	1	0	0	0	0
5	0	0	2	1	0	198	1	0	2	0	0	0	0	0	0	0	0	0	0	0	0	0	0	0	0	0	0	0	8	0	0	0	0	0	0	0
6	0	0	0	0	1	0	213	0	5	0	0	0	0	0	0	0	1	0	0	0	0	0	0	0	0	0	0	0	0	0	0	0	0	0	0	0
7	0	0	1	0	0	0	0	213	0	0	0	0	0	0	0	0	0	0	0	0	0	0	0	0	0	0	0	0	1	0	0	0	0	0	0	0
8	0	0	0	0	0	1	1	0	213	1	1	13	0	0	0	0	0	0	0	0	0	0	0	1	0	0	0	0	1	0	0	0	0	0	0	0
9	0	0	0	0	0	0	1	0	2	224	0	0	0	0	0	0	0	0	0	0	0	0	0	0	0	0	0	0	0	0	0	0	0	0	0	0
A	0	0	0	0	3	0	1	0	0	0	232	0	0	0	0	0	0	0	1	0	0	0	0	0	0	0	0	0	0	0	0	0	0	0	0	0
B	0	0	0	0	0	1	1	0	23	0	0	74	0	0	0	0	0	1	0	0	0	0	0	0	0	0	0	2	0	0	0	0	0	0	0	0
C	0	0	0	0	0	0	1	0	0	0	0	0	68	0	1	0	1	0	0	0	0	0	0	0	1	0	0	0	0	0	0	0	0	0	0	0
D	9	0	0	0	0	0	0	0	1	0	1	0	0	40	0	0	0	0	0	0	0	0	0	0	0	0	0	0	0	0	0	0	0	0	0	0
E	0	0	1	0	0	2	1	0	0	1	0	0	1	0	167	1	1	0	0	0	0	0	0	0	0	0	0	0	0	0	0	0	0	0	0	1
F	0	0	0	0	0	0	0	1	0	0	0	0	0	0	1	48	0	0	0	0	2	0	0	0	0	1	0	0	0	0	0	0	0	0	0	0
G	2	0	0	0	0	0	2	0	0	0	0	1	1	0	1	0	21	0	0	0	0	0	0	0	0	0	0	0	1	0	0	0	0	0	0	0
H	0	0	0	0	0	0	2	0	0	0	0	1	0	0	0	0	0	30	0	0	0	0	2	4	0	0	0	0	0	0	0	0	0	0	0	0
I	0	6	0	0	0	0	0	0	0	0	0	0	0	0	0	0	0	0	25	0	0	1	0	0	0	0	0	0	0	0	0	0	1	0	0	0
J	0	1	1	1	0	0	0	0	0	0	0	0	0	0	0	0	0	0	0	26	0	0	0	0	0	0	0	0	0	0	0	0	0	0	0	0
K	0	0	0	0	0	0	2	0	0	0	0	0	0	0	0	1	0	2	0	0	84	0	0	1	0	0	0	1	0	0	0	0	0	1	0	0
L	0	0	1	0	1	1	0	0	0	0	0	0	0	0	0	0	0	0	1	0	0	185	0	0	0	0	0	0	0	0	0	0	0	0	0	0
M	0	0	0	0	0	0	0	0	1	0	0	0	0	0	0	0	0	1	0	0	0	0	23	8	0	0	1	0	0	0	0	0	0	0	0	0
N	0	0	0	0	0	0	0	0	1	0	0	0	0	0	0	0	0	3	0	0	1	0	1	55	0	0	0	0	0	0	0	0	7	0	0	0
O	5	0	0	0	0	0	0	0	0	0	0	0	1	1	0	0	0	0	0	0	0	0	0	0	7	0	1	0	0	0	0	0	0	0	0	0
P	0	0	0	0	0	0	0	1	0	1	1	0	0	0	0	2	0	0	0	0	0	0	0	0	0	19	0	0	0	0	0	0	0	0	0	0
Q	5	0	0	0	0	0	0	0	1	1	0	0	0	1	0	0	1	0	0	0	0	0	0	0	1	0	3	0	0	0	0	0	0	0	0	0
R	0	0	0	0	0	0	2	0	3	1	1	1	0	0	1	0	0	0	0	0	2	0	0	0	0	0	0	47	0	0	0	0	0	0	0	0
S	0	0	3	1	0	6	1	0	0	0	0	0	0	0	0	0	0	0	0	0	0	0	0	0	0	0	0	0	37	0	0	0	0	0	0	0
T	0	0	0	0	0	0	0	1	0	0	0	0	0	0	0	0	0	0	0	0	0	0	0	0	0	0	0	0	0	32	0	0	0	0	0	0
U	0	0	0	0	0	0	0	0	0	0	0	0	1	0	0	0	1	0	0	0	0	0	0	0	0	0	0	0	0	0	27	0	0	0	0	0
V	0	0	0	0	0	0	0	0	0	0	0	0	0	0	0	0	0	0	0	0	2	0	0	0	0	0	0	0	0	0	0	24	0	0	1	0
W	0	0	0	0	0	1	0	0	0	0	0	0	0	0	0	0	0	0	0	0	0	0	0	12	0	0	0	1	0	0	0	0	23	0	0	0
X	0	1	2	1	0	0	0	0	0	0	0	0	0	0	0	0	0	0	0	0	0	0	0	0	0	0	0	0	0	0	0	0	0	39	0	0
Y	0	0	0	0	0	0	0	0	0	0	0	0	0	0	0	0	0	0	0	0	0	0	0	0	0	0	0	0	0	0	0	1	0	0	15	0
Z	0	0	5	0	0	0	0	0	0	0	0	0	0	0	0	0	0	0	0	0	0	0	0	0	0	0	0	0	0	1	0	0	0	0	0	42

**Table 5:** Confusion matrix of OCR using MLP classifier algorithm.

$\frac{\mathrm{Actual}\;\mathrm{Class}}{\mathrm{Predicted}\;\mathrm{Class}}$	0	1	2	3	4	5	6	7	8	9	A	B	C	D	E	F	G	H	I	J	K	L	M	N	O	P	Q	R	S	T	U	V	W	X	Y	Z
0	145	0	0	0	0	0	1	0	1	0	0	0	0	2	0	0	0	0	0	1	0	0	0	0	3	0	1	0	0	0	0	0	0	0	0	0
1	0	154	1	0	0	0	0	3	0	0	0	0	0	0	1	0	0	0	4	1	0	1	0	0	0	0	0	0	0	0	0	0	0	0	0	0
2	0	1	209	1	0	0	0	4	0	2	0	2	0	0	1	0	1	0	0	2	0	0	2	1	0	0	0	0	4	0	0	0	0	2	0	5
3	0	0	0	200	0	2	0	0	0	0	0	0	0	0	0	0	0	0	0	0	0	0	0	0	0	0	0	0	1	0	0	0	0	1	0	0
4	0	0	0	0	211	0	0	0	0	1	1	0	0	0	0	0	1	1	0	0	1	1	0	1	0	0	0	0	0	0	0	1	0	0	0	0
5	0	1	0	1	0	199	0	0	1	0	0	2	0	1	0	0	0	0	2	0	0	0	0	2	0	0	0	0	3	0	0	0	0	0	0	0
6	0	0	0	0	1	0	210	0	1	0	0	1	0	0	0	0	0	0	0	0	2	2	0	0	0	0	0	2	1	0	0	0	0	0	0	0
7	0	2	0	0	0	0	0	213	0	0	0	0	0	0	0	0	0	0	0	0	0	0	0	0	0	0	0	0	0	0	0	0	0	0	0	0
8	0	0	0	0	0	2	0	0	218	1	0	7	0	0	0	0	0	0	0	0	0	0	0	1	1	0	0	2	0	0	0	0	0	0	0	0
9	0	0	0	1	0	0	0	0	1	221	0	0	0	0	0	0	0	0	1	0	0	0	0	0	0	1	0	0	1	0	0	1	0	0	0	0
A	0	0	0	0	5	0	2	0	2	0	225	0	0	0	0	0	0	0	1	0	0	1	0	1	0	0	0	0	0	0	0	0	0	0	0	0
B	0	0	0	0	0	1	0	0	13	0	0	81	1	1	0	0	0	0	0	0	0	0	0	0	0	0	0	5	0	0	0	0	0	0	0	0
C	0	0	0	0	0	0	0	0	0	0	0	0	69	0	0	0	2	0	0	0	0	1	0	0	0	0	0	0	0	0	0	0	0	0	0	0
D	2	0	0	0	0	0	0	0	0	0	0	2	0	47	0	0	0	0	0	0	0	0	0	0	0	0	0	0	0	0	0	0	0	0	0	0
E	0	0	0	0	0	0	0	0	0	0	0	0	0	0	172	2	0	0	2	0	0	0	0	0	0	0	0	0	0	0	0	0	0	0	0	0
F	0	0	0	0	0	0	0	0	0	0	0	1	0	0	0	52	0	0	0	0	0	0	0	0	0	0	0	0	0	0	0	0	0	0	0	0
G	0	0	0	0	0	0	0	0	0	0	0	0	1	0	0	0	28	0	0	0	0	0	0	0	0	0	0	0	0	0	0	0	0	0	0	0
H	0	0	0	0	0	1	0	0	0	0	0	0	0	0	0	0	0	34	0	0	0	0	2	1	0	0	0	0	0	0	0	0	1	0	0	0
I	0	4	0	0	0	1	0	0	0	0	0	0	0	0	0	0	0	0	24	0	0	1	0	0	0	0	0	0	0	0	0	0	1	0	0	2
J	0	1	1	1	0	0	0	0	0	0	0	0	0	0	0	0	0	0	0	26	0	0	0	0	0	0	0	0	0	0	0	0	0	0	0	0
K	0	0	0	0	0	0	0	0	1	0	0	0	0	0	0	0	0	1	0	0	84	0	0	0	0	0	0	4	0	0	0	0	0	1	0	1
L	0	0	0	0	0	0	0	0	0	0	0	0	0	0	1	0	0	0	0	0	0	188	0	0	0	0	0	0	0	0	0	0	0	0	0	0
M	0	0	0	0	0	0	0	0	0	0	0	0	0	0	0	0	0	0	0	0	0	0	28	5	0	0	0	0	0	0	0	0	0	1	0	0
N	0	0	0	0	0	0	0	0	1	0	0	0	0	0	0	1	1	1	0	0	0	0	1	60	0	0	0	0	0	0	1	0	2	0	0	0
O	8	0	0	0	0	0	0	0	0	0	0	0	1	0	0	0	0	0	0	0	0	0	0	0	6	0	0	0	0	0	0	0	0	0	0	0
P	0	0	0	0	0	0	0	0	3	0	0	0	0	0	0	1	0	0	0	0	0	0	0	0	0 1	9	0	1	0	0	0	0	0	0	0	0
Q	1	0	0	0	0	0	0	0	1	1	0	0	0	0	0	0	0	0	1	0	0	0	0	0	2	0	6	0	0	0	1	0	0	0	0	0
R	0	0	0	0	0	1	0	0	0	0	1	0	1	0	0	1	0	0	0	0	3	0	1	0	0	0	0 4	9	0	0	0	1	0	0	0	0
S	0	1	1	3	0	3	0	0	1	0	0	0	0	0	0	0	1	0	0	0	0	0	0	0	0	0	0	0 3	7	0	0	0	0	1	0	0
T	0	1	0	0	0	0	0	1	0	0	0	0	0	0	0	0	0	0	0	0	0	0	0	0	0	0	0	0	0 3	1	0	0	0	0	0	0
U	0	0	0	0	0	0	0	0	0	0	0	0	0	0	0	1	0	0	0	0	0	0	0	0	0	0	0	0	0	0 2	8	0	0	0	0	0
V	0	0	0	0	0	0	0	0	0	0	0	0	0	0	0	1	0	0	0	0	0	0	0	0	0	1	0	0	0	0	0 2	3	0	0	2	0
W	0	0	0	0	1	0	0	0	0	0	0	0	0	0	0	0	0	0	0	0	1	0	2	1	0	0	0	0	0	1	0	0 3	1	0	0	0
X	0	0	0	1	0	0	0	0	0	0	0	0	0	0	0	0	0	0	0	0	0	0	0	0	0	0	0	0	0	0	0	0	0 4	1	0	1
Y	0	0	0	1	0	0	0	0	0	0	0	0	0	0	0	0	0	0	0	0	0	0	0	0	0	0	0	0	0	0	0	0	0	0 1	5	0
Z	0	1	3	0	0	0	0	1	0	0	0	0	0	0	1	0	0	0	0	0	0	0	0	0	0	0	0	0	0	1	0	0	0	2	0 3	9

**Table 6:** Performance comparison of time (Seconds) taken by the proposed system implemented on a PC (running Matlab) and a Raspberry Pi (Python + OpenCV).

	PC[Matlab]			Raspberry Pi (Python)		
$\frac{\mathrm{Characters}\;\mathrm{on}\;\mathrm{LP}}{\mathrm{Processing}\;\mathrm{on}\;\mathrm{LP}}$	**5 Characters**	**6 Characters**	**7 Characters**	**5 Characters**	**6 Characters**	**7 Characters**
Vehicle Detection and LP Extraction	3.2	3.19	3.21	0.13	0.11	0.14
LP Characters Segmentation	2.1	2.14	2.27	0.1	0.12	0.13
Characters Recognition and Barrier Control	3.22	3.26	3.37	0.16	0.17	0.21
Total Time Taken (Seconds)	8.52	8.59	8.85	0.39	0.4	0.48

## Dataset Availabity

Dataset will be provided on request by the corresponding author.
